# Reports of Cases at Fort Pitt Hospital

**Published:** 1859-04

**Authors:** George Williamson

**Affiliations:** Staff Surgeon


*Reports of Cases at Fort Pitt Hospital.* By GEORGE WILLIAMSON, M. D., Staff Surgeon.

*Fistula in Perineo.*—Private A. H——, aged nineteen; total service, two years at home. He was admitted into Regimental Hospital on the 28th of April, 1857, for hernia humoralis, consequent on gonorrhœa. The swelled testicle soon subsided, but was followed by inflammation along the course of the urethra, and two small abscesses formed in the neighborhood of the scrotum and perinæum, through which urine soon after commenced to flow. The urine continued to dribble through two fistulous openings, especially through the one farthest back, situated a little to the right side of the raphé. Various attempts had been made to heal these fistulæ, but without success.—His general health suffered from confinement to hospital, and, as he did not appear likely ever to become an efficient soldier, he was sent to Chatham, and was admitted on December 16th, 1857. There are now two fistulous openings on the right side of the raphé of the perinæum, one situated immediately behind the scrotum, and the other about an inch and a half posterior, through which urine escaped at all times. On inserting a catheter into the urethra, and a probe into the sinuses, the latter were forced to communicate with the former. The skin around the perinæum and lower part of the nates was irritated from the urine, and there were a few clusters of condylomata around the anus.

Jan. 8th.—A staff having been passed into the urethra, and a probe introduced into the fistulous opening, the two sinuses were found to communicate, and were laid into one; their connexion with the urethra was tried to be made out, and an incision was made upon the groove of the staff a quarter of an inch in extent, and the wound stuffed with lint to the bottom. A silver catheter (No. 6) was introduced and fixed with tapes, and the patient placed in bed.

31st.—The catheter is still kept in the urethra, from which there is some gonorrhœal discharge, with erections of the penis during the night, caused by the catheter. The wound in the perinæum is looking healthy and filling up.

March 1st.—The incision has almost closed, and no urine has come through it since the operation. He passes urine in a good stream, and the gonorrhœal discharge has ceased.

April 21st.—The wound in the perinæum has entirely closed, and the urine passes freely by the urethra and none by the perinæum. He is a stout, healthy young lad, and is discharged to join his regiment.

This case shows the effects of gonorrhœal inflammation extending along the urethra, causing perineal abscesses which ultimately communicate with the urethra; and the good effects of laying open the sinus in the perinæum, the introduction and retention of the catheter during the process of cure, and the stuffing of the wound to the bottom daily with dry lint.—*London Lancet.*

*Fort Pitt, Chatham,* 1858.

